# Hospital readmissions with acute infectious diseases in New Zealand children < 2 years of age

**DOI:** 10.1186/s12887-018-1079-x

**Published:** 2018-03-05

**Authors:** Silvia Seibt, Catherine A. Gilchrist, Peter W. Reed, Emma J. Best, Anthony Harnden, Carlos A. Camargo, Cameron C. Grant

**Affiliations:** 1 Paediatrics, Taranaki Base Hospital, New Plymouth, New Zealand; 2 Department of Paediatrics: Child and Youth Health, Faculty of Medical and Health Sciences, University of Auckland, Private Bag 92019, Wellesley Street, Auckland, 1142 New Zealand; 3 Children’s Research Centre, Starship Children’s Hospital, Auckland, New Zealand; 4 Infectious Diseases, Starship Children’s Hospital, Auckland District Health Board, Auckland, New Zealand; 5 Nuffield Department of Primary Care Health Sciences, University of Oxford, Oxford, England; 6 Emergency Medicine, Massachusetts General Hospital, Harvard Medical School, Boston, USA; 7 General Paediatrics, Starship Children’s Hospital, Auckland District Health Board, Auckland, New Zealand

**Keywords:** Infant, Child preschool, Cohort studies, Diarrhea, Hospitalization, Patient readmission, Respiratory tract infections, Skin and soft tissue infections, Urinary tract infections

## Abstract

**Background:**

Infectious diseases are the leading cause of hospital admissions in young children. Hospitalisation with an infectious disease is a recurrent event for some children. Our objective was to describe risk factors for infectious disease readmission following hospital admission with an infectious disease in the first two years of life.

**Methods:**

We performed a national cohort study of New Zealand children, born 2005–2009, with an infectious disease admission before age 24 months. Children readmitted with an infectious disease within 12 months of the first infectious disease admission were identified. Every infectious disease admission was categorised as a respiratory, enteric, skin and soft tissue, urinary or other infection. Independent associations of demographic and child health factors with infectious disease readmission were determined using multiple variable logistic regression.

**Results:**

From 2005 to 2011, there were 69,902 infectious disease admissions for 46,657 children less than two years old. Of these 46,657 children, 10,205 (22%) had at least one infectious disease readmission within 12 months of their first admission. The first infectious disease admission was respiratory (54%), enteric (15%), skin or soft tissue (7%), urinary (4%) or other (20%). Risk of infectious disease readmission was increased if the first infectious disease admission was respiratory (OR = 1.87, 95% CI 1.78–1.95) but not if it was in any other infectious disease category.

Risk factors for respiratory infectious disease readmission were male gender, Pacific or Māori ethnicity, greater household deprivation, presence of a complex chronic condition, or a first respiratory infectious disease admission during autumn or of ≥3 days duration. Fewer factors (younger age, male gender, presence of a complex chronic condition) were associated with enteric infection readmission. The presence of a complex chronic condition was the only factor associated with urinary tract infection readmission and none of the factors were associated with skin or soft tissue infection readmission.

**Conclusions:**

In children less than two years old, infectious disease readmission risk is increased if the first infectious disease admission is a respiratory infectious disease but not if it is another infectious disease category. Risk factors for respiratory infectious disease readmission are different from those for other infectious disease readmissions.

**Electronic supplementary material:**

The online version of this article (10.1186/s12887-018-1079-x) contains supplementary material, which is available to authorized users.

## Background

Globally, infectious diseases are a leading cause of morbidity and mortality in young children [1]. From 2004 to 2008 in New Zealand (NZ), infectious diseases accounted for 60% of hospitalisations of children < 5 years old [2]. Most infectious disease related hospitalisations were due to lower respiratory tract, enteric, and skin and soft tissue infections [2].

Hospitalisation rates for infectious diseases are higher in NZ than in other developed countries. Children < 2 years old are more than twice as likely to be hospitalised with bronchiolitis in NZ (2006–2010: 45/1000) than in England (2007–2010: 20/1000), or the United States (US) (2000–2009: 16/1000) [3–5]. Hospitalisation rates for pneumonia in children < 2 years old in NZ (2006–2010: 14/1000) are twice those in the US (2007–2009: 7/1000) [3, 6], and children < 1 year old have eight times the US rate of skin infection hospitalisation (NZ 2006–2010: 21/1000, US 2005 2.4/1000) [3, 7]. In recent decades the rate of infectious disease hospital admissions in NZ has increased [2]. This represents a true increase in disease incidence, attributed to ethnic and social inequalities, and to disparities in social determinants of health including household income, housing conditions, and access to healthcare [2].

Hospitalisation with an infectious disease (ID) is a recurrent event for some children. In a US study, the 3% of children with four-or-more recurrent admissions, including but not limited to ID, accounted for 19% of all paediatric hospital admissions [8]. Identifying strategies to prevent readmission is therefore an important component of quality of care improvement [9].

Early identification of children at risk of ID readmission and prevention of repeated illness episodes may prevent subsequent chronic disease. For example, globally almost two-thirds of children with ID-associated bronchiectasis have a history of recurrent acute lower respiratory infections (ALRIs) [10–12]. Bronchiectasis is particularly prevalent in indigenous children from NZ, Australia, Alaska, and Canada and a history of early infant pneumonia and/or recurrent ALRIs is associated with bronchiectasis in over 90% of cases [11, 13, 14].

Our aim was to describe recurrent hospitalisations for infectious diseases in young NZ children, including factors associated with ID readmission. The ability to identify children at risk of recurrent disease when they first present to hospital allows the development and evaluation of interventions targeted to this at-risk group.

## Methods

### Study design and setting

We created a national cohort study of NZ children born over a five-year period (2005–2009) and described their ID hospitalisations. We identified the children hospitalised with an ID during their first 24 months of life, and the subset of children subsequently readmitted with an ID within 12 months of their initial ID hospitalisation were enrolled in the study. The study protocol was reviewed by the NZ Health and Disabilities Ethics Committee, with the ethics committee determining that ethical approval was not required. The NZ Ministry of Health granted data access, providing data with encrypted NHI numbers to maintain patient anonymity. The Ministry of Health did not grant permission for data sharing.

### Study population and sample

Children were eligible if born between 1st January 2005 and 31st December 2009 and a NZ resident at the time of their first ID hospitalisation. Hospitalisation data were collected over six years, from 1st January 2005 to 31st December 2011. Children were excluded if they had an ID admission in their first 24 months of life but died within 12 months of this first admission (*n* = 117). Permission to access data on cause of death was not obtained.

A hospital admission was defined as an overnight stay in a hospital inpatient ward. We excluded episodes where a child was admitted and discharged on the same day. In NZ all acute ID hospital admissions during childhood are to public hospitals.

Hospitalisation data were obtained from the NZ national dataset of hospital admissions, the National Minimum Dataset (NMDS) [15]. We used the national health index (NHI) number, a unique identifier assigned to every person on contact with health services in NZ, to identify all hospitalisations for each child. To maintain anonymity, data provided from the NMDS contained an encrypted unique identifier for each child. Thus, written informed consent was not required.

### Identification of infectious disease hospital admissions

Using the approach developed by the US Centers for Disease Control and Prevention [16, 17], and previously used in NZ [2], we defined an ID hospitalisation as one for which the principal International Classification of Disease (ICD-10) code was for an ID (Additional file 1).

**Table 1:** Demographic and illness characteristics of children aged less than 2 years admitted to hospital with an infectious disease from 1st January 2005 to 31st December 2011

	Children admitted to hospital with an infectious disease before age 2 years
Variable	*n* = 46,540
Demographic characteristics	
Age in months, median (IQR)	7.2 (2.4–13.2)
Age, *n* (%)	
Less than 6-months-old	19,028 (41)
6- to 23-months-old	27,512 (59)
Gender, *n* (%)	
Male	26,038 (56)
Female	20,502 (44)
Ethnicity^a^, *n* (%)	
Pacific	7902 (17)
Māori	15,876 (34)
Asian	2499 (5)
Other	740 (2)
European	19,411 (42)
Household deprivation^b^, *n* (%)	
Dep 9 & 10 (most deprived)	18,575 (40)
Dep 7 & 8	10,996 (24)
Dep 5 & 6	7226 (16)
Dep 3 & 4	5171 (11)
Dep 1 & 2 (least deprived)	4435 (10)
Season of admission^c^, *n* (%)	
Autumn	8586 (18)
Winter	17,285 (37)
Spring	13,162 (28)
Summer	7507 (16)
Illness characteristics	
Length of stay in days, median (25th, 75th centile)	3 (2, 4)
Diagnostic group of first admission, *n* (%)	
Acute respiratory infection (ARI)	25,256 (54)
Enteric infection	6909 (15)
Skin and soft tissue infection (SSTI)	3210 (7)
Urinary tract infection (UTI)	1826 (4)
Other	9339 (20)
Presence of complex chronic conditions, *n* (%)	
All complex chronic conditions	894 (2)
Neuromuscular	45 (0.1)
Cardiovascular	403 (0.9)
Respiratory	116 (0.2)
Renal	43 (0.1)
Gastrointestinal	78 (0.2)
Haematologic and immunologic	38 (0.1)
Metabolic	3 (0.01)
Other congenital or genetic defect	133 (0.3)
Malignancy	35 (0.1)

^a^Ethnicity not stated, *n* = 112

^b^Area-level socio-economic deprivation was measured using the NZ Index of Deprivation (NZDep06), grouped into quintiles [18]. Data were missing for 137 (0.2%) children

^c^Autumn = March to May; Winter = June to August; Spring = September to November; Summer = December to February

*CI* Confidence interval

*IQR* Interquartile range

Within the NMDS, each hospital admission may include more than one inpatient event. For example, a child admitted to intensive care and then transferred to the inpatient ward is described as two separate events. To ensure each admission was counted only once, consecutive events with the same discharge and admission dates were counted as a single hospitalisation.

### Study sample demographics and illness history

Demographic information was obtained from the NMDS. Ethnicity (European, Māori, Pacific, Asian and Other) was defined as that stated by parents at the first admission. Socioeconomic status was defined using a small area unit level descriptor of household deprivation created from national census data (NZDep2006) grouped as quintiles [18]. As defined by Berry et al. (2011), children with complex chronic conditions (CCC) known to be associated with recurrent hospital admission were identified from the ICD-10 codes listed for the first ID admission [8].

### Statistical analyses

ID hospitalisations were grouped into five diagnostic categories: acute respiratory infection (ARI), enteric infection, skin or soft tissue infection (SSTI), urinary tract infection (UTI) and other ID (Additional file 1). Other ID included septicaemia, meningococcal disease, meningitis, osteomyelitis, and septic arthritis. An ID readmission was defined as a second or subsequent hospital admission with an ID principal discharge code, > 1 day after the discharge from the first admission and < 12 months from the original ID admission.

**Table 2:**
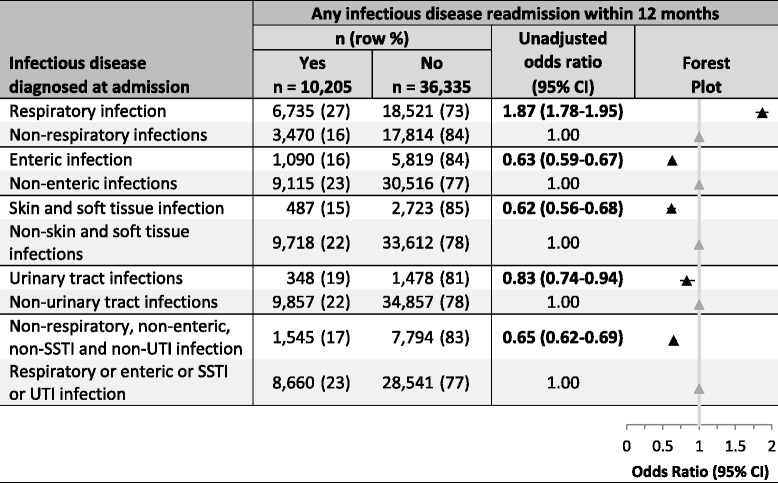
Risk of any infectious disease readmission within a 12 month period based upon infectious disease discharge diagnosis category at first admission

*CI* Confidence interval, *SSTI* Skin or soft tissue infection, *UTI* Urinary tract infection

Data were analysed using JMP V10.0 (SAS Inc.). We used multivariable logistic regression to identify independent associations between demographic variables and the risk of hospital readmission with an ID. We determined whether the risk of readmission with an ID varied by type of ID causing the first admission (ARI, enteric, SSTI, UTI or other ID), and, within the ARI group, by type of acute lower respiratory infection (pneumonia, bronchiolitis, influenza and other ARI) (Additional file 2). Within the ARI, enteric, SSTI, UTI or other ID diagnostic groups, we then described the risk factors associated with readmission with a second ID episode within the same ID diagnostic group. Associations were reported using adjusted odds ratios (OR) and 95% confidence intervals (CI).

**Table 3:**
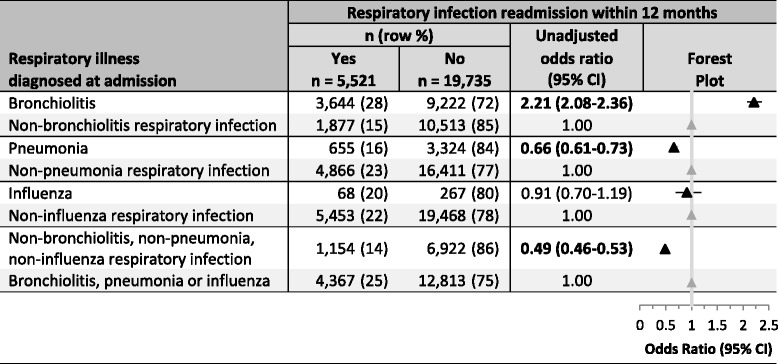
Risk of respiratory disease readmission within a 12 month period based upon respiratory illness present at the first admission

*CI* Confidence interval

## Results

### Study population and sample

**Fig. 1:**
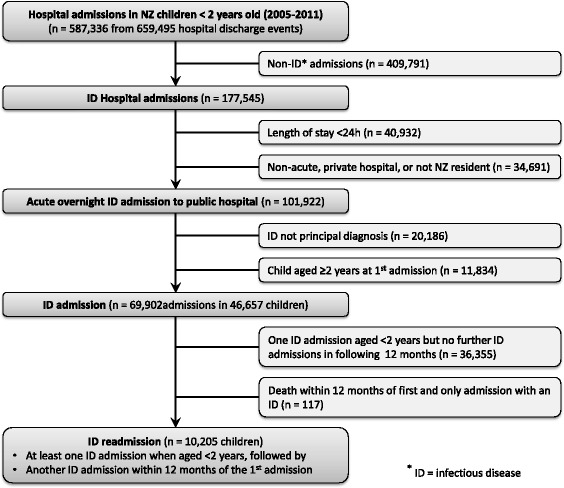
Flow diagram describing study design

There were 659,495 hospital discharge events (from 1st January 2005 and 31st December 2011) for children born between 1st January 2005 and 31st December 2009 (Fig. 1). These events, which included formal transfers and other patient movements, converted to 587,336 hospital admissions, of which 177,545 (30%) included an ID discharge code. We excluded 34,691 admissions (non-acute, to a private hospital or not a NZ resident); and 40,932 admissions lasting < 24 h. Of the 101,922 acute overnight public hospital ID admissions, for 20,186 the ID discharge code was not the principal code, and for 11,834 the first ID admission occurred at age ≥ 2 years.

**Table 4:**
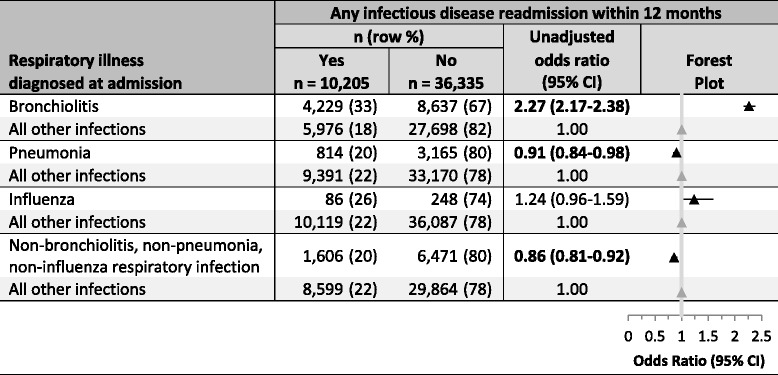
Risk of any infectious disease readmission within a 12 month period based upon respiratory illness present at the first admission

*CI* Confidence interval

There were 69,902 ID hospital admissions for 46,657 children < 2 years old, of whom 10,205 (22%) had at least one subsequent ID readmission within the 12 months following their first admission. Therefore, 15% of the national birth cohort from 2005 to 2009 had an ID hospital admission before age two years, with 3% having at least one further ID admission within the next 12 months.

### Characteristics of children hospitalised with an infectious disease (Table 1)

The median (interquartile range (IQR)) age at first ID admission was 7.2 (2.4–13.2) months. Compared with the ethnicity of live births in New Zealand in 2010 (27% Māori, 11% Pacific, 11% Asian, and 51% European/other), there were higher percentages of Māori (34%, *p* < 0.001) and Pacific (17%, *P* < 0.001) children among those hospitalised with an ID. By contrast, there were lower percentages of Asian (5%, *P* < 0.001) and European/other (43%, *P* < 0.001) children hospitalised with an ID [19].

Of the 46,540 children hospitalised with an ID, 18,575 (40%) lived in the most deprived households and 894 (2%) had an underlying CCC. The most frequent season of first ID admission was winter (17,285/46,540 37%) and the majority of first admissions were for an ARI (25,256/46,540 54%).

### Infectious disease group and readmission risk (Table 2)

Readmission occurred at a median (IQR) of 77 (26–177) days after the first ID admission. Median (IQR) days to ID readmission was 67 (25–157) if the first admission was ARI, 96 (23–198) if it was enteric infection, 100 (34–195) if it was an SSTI and 115 (45–202) if it was a UTI.

Overall, 6735/10,205 (66%) of the ID readmissions were with an ARI. In comparison with all other infections, children whose first ID admission was an ARI were at increased risk (OR = 1.87, 95% CI 1.78–1.95) of readmission, whereas those whose first ID admission was an enteric infection (OR = 0.63, 0.59–0.67), SSTI (OR = 0.62, 95% CI 0.56–0.68), UTI (OR = 0.83, 95% CI 0.74–0.94) or other infection (OR = 0.65, 95% CI 0.62–0.69) were at decreased risk.

### Acute respiratory infection group and readmission risk (Tables 3 and 4)

Among children whose first admission was an ARI, the risk of readmission with an ARI was increased if this first admission was with bronchiolitis (OR = 2.21, 95% CI 2.08–2.36) and decreased if the first admission was with pneumonia (OR = 0.66, 95% CI 0.61–0.73), or an ARI that was not bronchiolitis, pneumonia, or influenza (OR = 0.49, 95% CI 0.46–0.53) (Table 3). Among children whose first admission was an ARI, the principal discharge diagnosis of the readmission was lower ARI in 82%. In comparison with those whose first ID admission was an upper ARI, children whose first ID admission was a lower ARI were at increased risk of readmission with any ID (29% vs. 19%, OR = 1.75, 95% CI 1.63–1.87).

Among children whose first admission was with an ARI, the risk of readmission with any ID was increased if this first admission was with bronchiolitis (OR = 2.27, 95% CI 2.17–2.38) and decreased if the first admission was with pneumonia (OR = 0.91, 95% CI 0.84–0.98), or an ARI that was not bronchiolitis, pneumonia, or influenza (OR = 0.86, 95% CI 0.81–0.92) (Table 4).

### Factors associated with readmission with an acute respiratory, enteric, skin or soft tissue, or urinary tract infection

Of the 25,256 children whose first ID admission was ARI, readmission with an ARI occurred in 5521 (22%), with median (IQR) time to readmission of 57 (24–134) days (Additional file 3). Of the 6090 children whose first ID admission was an enteric infection, readmission with an enteric infection occurred in 449 (6%; median time to readmission 39 (5–145) days) (Additional file 4). Of the 3210 children admitted with an SSTI, readmission with an SSTI occurred in 205 (9%; median time to readmission 54 (16–153) days) (Additional file 5). Of the 1826 children admitted with a UTI, readmission with a UTI occurred in 101 (6%; median time to readmission 115 (45–202) days) (Additional file 6).

Children at increased risk of a second ARI admission were of younger age when first admitted with an ARI (age < 6 months vs. 6 to 23 months old, OR = 1.62, 95% CI 1.52–1.73), male gender (OR = 1.20, 95% CI 1.13–1.28), Pacific (OR = 2.14, 95% CI 1.95–2.36) or Māori (OR = 1.98, 95% CI 1.82–2.14) versus European ethnicity, or living in the most (OR = 1.31, 95% CI 1.14–1.51) or second most (OR = 1.21, 95% CI 1.05–1.40) deprived compared with the least deprived quintile of households. The risk of an ARI readmission increased (OR = 1.14, 95% CI 1.01–1.29) for children whose first ARI hospital admission was in autumn compared with summer. Children with a CCC (OR = 3.25, 95% CI 2.73–3.87) or whose first ARI admission was ≥3 days in duration (OR = 1.53, 95% CI 1.43–1.63) were at increased risk of an ARI readmission (Additional file 3).

In contrast with ARIs, demographic and illness factors were less consistently associated with the risk of readmission with a second enteric infection, SSTI or UTI (Additional files 4, 5 and 6). Children at increased risk of a second enteric infection admission were those aged < 6 months (OR = 1.53, 95% CI 1.24–1.87) when first admitted with an enteric infection, of male gender (OR = 1.25, 95% CI 1.03–1.52) or with a CCC (OR = 3.58, 95% CI 2.03–6.01) (Additional file 4). Children at increased risk of a second UTI were those with a CCC (OR = 3.49, 95% CI 1.45–7.51) (Additional file 6). None of these factors was associated with the risk of a second SSTI (Additional file 5).

## Discussion

In this national study, 15% of children in the 2005–2009 NZ birth cohort were hospitalised with an ID in the first two years of life, 40% of whom lived in the most deprived 20% of households. Twenty-two percent of these children (3% of the birth cohort) had at least one further ID admission within 12 months of their first admission. Two-thirds of the first ID admissions were for an ARI and over one-quarter of these children had a subsequent ARI admission. In contrast, a first ID admission for an enteric ID, SSTI or UTI was not associated with an increased risk of readmission within the same ID category.

In multivariable models, the risk factors associated with ARI readmission were younger age, male gender, Pacific or Māori ethnicity, living in a more deprived household, a first ARI admission during autumn, the presence of a CCC, or a first ARI admission of ≥3 days duration. Only younger age, male gender and presence of a CCC were associated with an increased risk of enteric ID readmission, and only presence of a CCC with an increased risk of UTI readmission. None of these factors were associated with the risk of SSTI readmission.

A strength of our study was the ability to describe ID hospital admissions for the national birth cohort. In NZ, discharge data for admissions to public hospitals are loaded into the NMDS within 21 days of discharge. We are confident, therefore, that our description of ID admissions is complete. By studying a five-year birth cohort (2005–2009) we minimised the effect of an isolated epidemic, for example that caused by the 2009 H1N1 influenza pandemic [20].

By limiting to ID principal discharge codes, our study underestimated the total number of ID admissions. However, limitation to principal codes was necessary to clearly describe the relationship between first and subsequent ID admissions. Our analyses were restricted to NMDS data and therefore the influence of other factors, such as cigarette smoking, or primary care received, was not evaluated, as they are not included in a national data set.

Our case definition was limited to overnight hospital admissions. This excludes very short-term hospital admissions, which may be more likely to occur due to systemic failure in the delivery of acute care in primary care and emergency departments [21].

Children with CCC and children supported with medical technology, for example a tracheostomy or ventriculo-peritoneal shunt, are especially vulnerable to events requiring hospital admission [8]. Whilst children with an underlying CCC were at an increased risk of ID readmission, in our study they accounted for only 2% of all of the children hospitalised with an ID before age 2 years.

Our data suggest that identification of children at risk of recurrent admission and intervention during the first admission may reduce total ID hospitalisation burden. Our study findings imply that such strategies should focus on preventing ARI readmission, given that the risk of ID readmission was increased for ARI but not for enteric infections, SSTIs or UTIs.

Providing specialist health care outside of the hospital is one such potential strategy. In England, the concept of “Hospital-at-Home” with paediatric nurse home care visits accessible 24 h per day was recently evaluated as an alternative to inpatient care for children with breathing difficulties, diarrhoea and vomiting, or fever [22]. Whilst the hospital-at-home care was well accepted by families, it did not result in any reduction in risk of hospital readmission in the subsequent 90 days [22].

Clinical pathways including specific admission and discharge criteria can help to reduce the rate of readmission within two weeks of the first admission, as shown in a recent Australian study of children < 12 months old hospitalised with bronchiolitis [23]. This clinical pathway included improved discharge planning, including specific discharge criteria, a discharge plan developed in consultation with parents, and communication with the primary care physician [23].

Can ARI readmissions over the longer term be prevented, beyond those due to the same respiratory illness that caused the first admission? A recent meta-analysis of 25 randomised controlled trials, which enrolled 11,321 participants aged 0 to 95 years, showed that vitamin D supplementation prevents ARIs [24]. Supplementation is of most benefit to those who are vitamin D deficient, and when daily or weekly vitamin D dosing regimens without bolus doses are used [24]. In Auckland, daily vitamin D_3_ supplementation during pregnancy and infancy was shown to reduce the proportion of children making a primary care ARI visit up to age 18 months [25]. Therefore, vitamin D_3_ supplementation following ARI hospital admission could potentially prevent ARI readmissions. We are currently conducting a randomised clinical trial to determine if vitamin D supplementation prevents ARI healthcare visits in children under two-years-old (Trial ID: ACTRN12616000659404).

Hospital admission also provides an immunisation update opportunity. Children with more acute illness visits are at increased risk of delayed immunisations [26]. The pneumococcal conjugate vaccine prevents a proportion of ARI hospital admissions, including a spectrum of ARI without radiographic evidence of pneumonia [27]. Our study shows that infants who present in autumn are more likely to represent with ARI. Hence offering seasonal influenza vaccination prior to hospital discharge, provided the child does not have a temperature > 38 °C [28], may reduce the risk of the winter readmission of these children.

Children of Māori and Pacific ethnicity and those living in deprived households continue to be at increased risk of hospital admission and readmission with infectious diseases [2]. Characteristics of the child’s home environment, such as overcrowding, dampness and mould, can increase the risk of contracting and being hospitalised with an ARI [29–32]. A recent NZ study found that the risk of ARI hospitalisation before age five years was increased for children living in households where there was gas heating in the bedroom the child slept in during their first year of life [33]. Thus, identification, during the first ARI admission, of the type of heating used in the child’s household and replacing gas heating with electric heating, particularly in the room where the child sleeps, could prevent ARI readmissions.

In summary, children hospitalised with an ARI in the first 2 years of life, and in particular a lower ARI, are a group for whom strategies are required to reduce the risk of ARI readmission. Contemporary evidence indicates potential interventions including vitamin D supplementation, ensuring immunisation status is up to date and replacing gas heating in the child’s bedroom. Randomised controlled trials of these interventions are necessary to determine which, if any, are sufficiently cost-effective for general implementation.

## Conclusions

Hospitalisation rates for infectious diseases continue to be high for NZ children. Of the 15% of children hospitalised with an infectious disease before age two years, one-in-five will have an infectious disease readmission within 12 months of their first admission. The risk of readmission is increased when the first infectious disease is respiratory, but not when it is an enteric, skin or urinary tract infection. Strategies to prevent infectious disease readmission should focus on children hospitalised prior to age two years with lower respiratory infections.

## Additional files

Additional file 1: Organ system, infectious disease diagnostic groups and associated ICD-10 codes [2, 34]. (DOCX 30 kb)

Additional file 2: Acute lower respiratory infection syndromes and associated ICD-10 codes [2, 34]. (DOCX 24 kb)

Additional file 3: Associations of demographic and illness characteristics with risk of hospital readmission with a second acute respiratory infection within 12 months of a first hospital admission with an acute respiratory infection. (DOCX 90 kb)

Additional file 4: Associations of demographic and illness characteristics with risk of hospital readmission with a second enteric infection within 12 months of a first hospital admission with an enteric infection. (DOCX 86 kb)

Additional file 5: Associations of demographic and illness characteristics with risk of hospital readmission with a second skin and soft tissue infection within 12 months of a first hospital admission with a skin and soft tissue infection. (DOCX 91 kb)

Additional file 6: Associations of demographic and illness characteristics with risk of hospital readmission with a second UTI within 12 months of a first hospital admission with a UTI. (DOCX 75 kb)

## Abbreviations

ARI: Acute Respiratory Infection
CCC: Complex Chronic Condition
CI: Confidence Interval
ICD-10: International Classification of Disease-10
ID: Infectious Disease
IQR: interquartile range
NHI: National Health Index
NMDS: National Minimum Dataset
NZ: New Zealand
OR: Odds Ratio
SSTI: Skin or Soft Tissue Infection
US: United States
UTI: Urinary Tract Infection
