# Supplementary material for: Hospital readmissions with acute infectious diseases in New Zealand children < 2 years of age
Source: BMC Pediatr. 2018 Mar 5;18:98. doi: 10.1186/s12887-018-1079-x (PMC5838880; doi:10.1186/s12887-018-1079-x)
Supplement: Supplementary file 1 — Organ system, infectious disease diagnostic groups and associated ICD-10 codes [2, 34]. (DOCX 30 kb) [file 12887_2018_1079_MOESM1_ESM.docx]

# Additional File 1: Organ system, infectious disease diagnostic groups and associated ICD-10 codes [[1](#_ENREF_1), [32](#_ENREF_32)].

| **Organ system** | **Infectious Disease Group** | **ICD-10-AM Codes** |
| --- | --- | --- |
| **Respiratory** | Ear infections | H600, H601, H602, H603, H608, H609, H62, H65, H66, H67, H680, H70, H730, H750, H830, H940 |
|  | Upper respiratory tract infection | J00, J01, J02, J03, J04, J05, J06, J32, J340, J36, J37, J390, J391 |
|  | Acute lower respiratory tract infection | A481, A482, B59, J09, J10, J11, J12, J13,J14, J15, J16, J17, J18, J20, J21, J22 |
|  | Chronic lower respiratory tract infection | J40, J41, J42, J440, J47, J85, J86, J988 |
|  | Tuberculosis | A15, A16, A17, A18, A19, N740, N741, J65 |
| **Enteric** | Enteric infections | A00, A01, A02, A03, A04, A05, A06, A07,A08 |
|  | Enteric symptoms | A09, I880, K528, K529, R11 |
|  | Gastrointestinal tract infections | K230, K231, K25, K26, K27, K28, K293, K294, K295, K35, K36, K37, K61, K630,K632, K650, K678, K908, K930 |
| **Skin and soft tissue** | Skin infections, typical | A46, L00, L01, L02, L03, L04, L050, L08 |
|  | Eye infections | B30, H000, H03, H043, H050, H100, H102, H103, H109, H130, H131, H160, H190, H191, H192, H220, H440, H451 |
|  | Breast infections | N61 |
|  | Infections of other anatomical sites | H00.0, H60.0, H60.1, H60.2, H60.3, H62.0,H62.4, J34.0, K61.0,H05.0, N48.2, N49.2N49.9, N76.4, A46 |
|  | Infected/unspecified/other dermatitis | L30.3, L30.8,L30.9 |
|  | Insect/spider bites | S10.13, S10.83, S10.93, S20.13, S20.33, S20.43, S20.83, S30.83S30.93, S40.83, S 50.83, S60.83, S70.83, S80.83, S90.83,T09.03, T11.08, T13.03,T14.03, T63.3, T63.4, T00.9 |
|  | Post traumatic/open wound infection | T79.3, T89.01, T89.02 |
|  | Scabies | B86 |
|  | Varicella with other complications | B01.8 |
| Urinary tract | Urinary tract infections | N300, N341, N351, N37, N390 |
| Other infectious disease | Septicaemia | A40, A41 |
|  | STI | A50, A51, A52, A53, A54, A55, A56, A57, A58, A59, A60, A63, A64, N290 |
|  | HIV/AIDS | B20, B21. B22, B23, B24 |
|  | Meningococcal Disease | A39 |
|  | Central nervous system viral infections | A801, A802, A803, A804, A809, A811, A812, A818, A819, A82, A83, A84, A85, A86, A87, A88, A89 |
|  | Central nervous system general infections | G00, G01, G02, G030, G039, G04, G05,G06, G07, G08, G09, G610 |
|  | Heart and circulatory infections | B332, I00, I01, I02, I05, I06, I07, I08, I09,I301, I33, I38, I39, I400, I410, I411, I412, I430, I716, I790, I791 |
|  | Oral infections | K02, K044, K046, K050, K052, K053, K113, K122 |
|  | Hepatic infections | K750, K770, K830 |
|  | Viral hepatitis | B15, B16, B17, B18, B19 |
|  | Kidney infections | N00, N05, N10, N136, N151 |
|  | Reproductive system infections, male | N410, N411, N412, N413, N431, N45, N481, N482, N490, N49, N51 |
|  | Reproductive system infections, female | N70, N71, N72, N73, N74, N751, N764,N87 |
|  | Osteomyelitis | M462, M463, M464, M465 |
|  | Joint infections | M00, M01 |
|  | Connective tissue infections | M021, M023, M03, M600, M630, M631, M632, M650, M651, M680, M710, M711, M896 |
|  | Neoplasms from infection | C11, C161, C162, C163, C164, C165, C166, C168, C169, C210, C211, C220, C46, C53, D002, D013, D06 |
|  | Postoperative Infections | T802, T814, T826, T827, T835, T836, T845, T846, T847, T857, T874 |
|  | Adverse effect of infectious disease treatment | R761, R762, T36, T37, T485, T487, T490, T495, T496, T499, T788, T789, T880, T881, T887 |
|  | Other bacterial Infections | A20, A21, A22, A23, A24, A25, A26, A27, A28, A30, A31, A32, A33, A34, A35, A36, A37, A38, A42, A43, A44, A480, A483, A484, A488, A49, A65, A66, A67, A68, A69, A70, A71, A74, A75, A77, A78, A79, B95, B96 |
|  | Other viral infections | A90, A91, A92, A93, A94, A95, A96, A98, A99, B00, B01, B02, B03, B04, B05, B06, B07, B08, B09, B25, B26, B27, B33, B34, B97 |
|  | Other mycoses | B35, B36, B37, B38, B39, B40, B41, B42, B43, B44, B45, B46, B47, B48, B49 |
|  | Other protozoan infections | B50, B51, B52, B53, B54, B55, B56, B57, B58, B60, B64 |
|  | Other infectious diseases | B65, B66, B67, B68, B69, B70, B71, B72, B73, B74, B75, B76, B77, B78, B79, B80, B81, B82, B83, B85, B87, B88, B89, B94, B99, E033, E321, F024, F071, I88, T64 |
|  | Infections of pregnancy and puerperium | O030, O035, O050, O055, O060, O065, O070, O075, O080, O040, O045,, O23, O411, O753, O85, O86, O9100, O9110, O9120, O98 |
|  | Perinatal infections | P002, P027, P23, P35, P36, P37, P38, P39 |
