# Supplementary material for: Hospital readmissions with acute infectious diseases in New Zealand children < 2 years of age
Source: BMC Pediatr. 2018 Mar 5;18:98. doi: 10.1186/s12887-018-1079-x (PMC5838880; doi:10.1186/s12887-018-1079-x)
Supplement: Supplementary file 2 — Acute lower respiratory infection syndromes and associated ICD-10 codes [2, 34]. (DOCX 24 kb) [file 12887_2018_1079_MOESM2_ESM.docx]

# Additional file 2: Acute lower respiratory infection syndromes and associated ICD-10 codes [[1](#_ENREF_1), [32](#_ENREF_32)].

| **Lower respiratory syndrome** | **Discharge diagnostic codes** |
| --- | --- |
| **Pneumonia** | J12 Viral pneumonia, not elsewhere classified  J13 Pneumonia due to *Streptococcus pneumoniae*  J14 Pneumonia due to *Haemophilus influenzae*  J15 Bacterial pneumonia, not elsewhere classified  J16 Pneumonia due to other infectious organisms, not elsewhere classified  J17 Pneumonia in diseases classified elsewhere  J18 Pneumonia, organism unspecified  J85 Abscess of lung and mediastinum  J86 Pyothorax |
| **Bronchiolitis** | J21 Acute bronchiolitis |
| **Influenza** | J09 Influenza due to certain identified influenza virus  J10 Influenza due to identified influenza virus  J11 Influenza, virus not identified |
| **LRTI neither pneumonia nor bronchiolitis nor influenza** | A481 Legionnaires disease  A482 Nonpneumonic Legionnaires disease [Pontiac fever]  B59 Pneumocytosis  J20 Acute bronchitis  J22 Unspecified acute lower respiratory infection  J40 Bronchitis, not specified as acute or chronic  J41 Simple and mucopurulent chronic bronchitis  J42 Unspecified chronic bronchitis  J440 Chronic obstructive pulmonary disease with acute lower respiratory infection  J47 Bronchiectasis  J988 Other specified respiratory disorders |
