# Supplementary material for: Hospital readmissions with acute infectious diseases in New Zealand children < 2 years of age
Source: BMC Pediatr. 2018 Mar 5;18:98. doi: 10.1186/s12887-018-1079-x (PMC5838880; doi:10.1186/s12887-018-1079-x)
Supplement: Supplementary file 3 — Associations of demographic and illness characteristics with risk of hospital readmission with a second acute respiratory infection within 12 months of a first hospital admission with an acute respiratory infection. (DOCX 90 kb) [file 12887_2018_1079_MOESM3_ESM.docx]

# Additional file 3. Associations of demographic and illness characteristics with risk of hospital readmission with a second acute respiratory infection within 12 months of a first hospital admission with an acute respiratory infection.

|  | **Respiratory infectious disease readmission within 12 months** | | | | |
| --- | --- | --- | --- | --- | --- |
|  | **n (row %)** | | **Multivariable** |  |  |
|  | **Yes** | **No** | **odds ratio** |  |  |
| **Variable** | **n = 5,521** | **n = 19,735** | **(95% CI)** | **Forest Plot** | ***P*-value** |
| **Demographic characteristics** | | | | | |
| **Age** |  |  |  |  |  |
| Less than 6 months | 2,789 (28) | 7,097 (72) | **1.62 (1.52-1.73)** |  | <0.001 |
| 6 to 23 months old | 2,732 (18) | 12,638 (82) | 1.00 |  |  |
| **Gender** |  |  |  |  |  |
| Male | 3,381 (23) | 11,236 (77) | **1.20 (1.13-1.28)** |  | <0.001 |
| Female | 2,140 (20) | 8,499 (80) | 1.00 |  |  |
| **Ethnicity*** |  |  |  |  |  |
| Pacific | 1,387 (29) | 3,477 (71) | **2.14 (1.95-2.36)** |  | <0.001 |
| Māori | 2,651 (27) | 7,285 (73) | **1.98 (1.82-2.14)** |  | <0.001 |
| Asian | 126 (12) | 901 (88) | 0.83 (0.68-1.01) |  | 0.06 |
| Other | 54 (17) | 270 (83) | 1.11 (0.81-1.49) |  | 0.51 |
| European | 1,294 (14) | 7,761 (86) | 1.00 |  |  |
| **Household deprivation^†^** |  |  |  |  |  |
| Dep 9 & 10 (most deprived) | 2,874 (26) | 8,207 (74) | **1.31 (1.14-1.51)** |  | **<0.001** |
| Dep 7 & 8 | 1,264 (21) | 4,638 (79) | **1.21 (1.05-1.40)** |  | **0.008** |
| Dep 5 & 6 | 667 (18) | 3,018 (82) | 1.11 (0.95-1.30) |  | 0.18 |
| Dep 3 & 4 | 408 (16) | 2,090 (84) | 1.08 (0.91-1.27) |  | 0.38 |
| Dep 1 & 2 (least deprived) | 295 (15) | 1,720 (85) | 1.00 |  |  |
| **Season of first admission**^ǂ^ |  |  |  |  |  |
| Autumn | 877 (25) | 2,700 (75) | 1.14 (1.01-1.29) |  | 0.04 |
| Winter | 2,607 (22) | 9,436 (78) | **0.84 (0.76-0.93)** |  | 0.001 |
| Spring | 1,449 (20) | 5,632 (80) | **0.80 (0.72-0.90)** |  | <0.001 |
| Summer | 588 (23) | 1,967 (77) | 1.00 |  |  |
| **Illness characteristics** | | | | | |
| **Presence of complex chronic condition** |  |  |  |  |  |
| Yes | 282 (49) | 294 (51) | **3.25 (2.73-3.87)** |  | <0.001 |
| No | 5,239 (21) | 19,441 (79) | 1.00 |  |  |
| **Length of stay** |  |  |  |  |  |
| ≥3 days | 3,718 (26) | 10,645 (74) | **1.53 (1.43-1.63)** |  | <0.001 |
| 2 days or less | 1,803 (17) | 9,090 (83) | 1.00 |  |  |
|  | | | | | |
| * Ethnicity not stated, n = 50  ^†^ Area-level socio-economic deprivation was measured using the NZ Index of Deprivation (NZDep06), grouped into quintiles [[13](#_ENREF_13)]. Data were missing for 137 (0.2%) children.  ^ǂ^ Autumn = March to May; Winter = June to August; Spring = September to November; Summer = December to February.  CI – confidence interval | | | | | |
