# Supplementary material for: Hospital readmissions with acute infectious diseases in New Zealand children < 2 years of age
Source: BMC Pediatr. 2018 Mar 5;18:98. doi: 10.1186/s12887-018-1079-x (PMC5838880; doi:10.1186/s12887-018-1079-x)
Supplement: Supplementary file 4 — Associations of demographic and illness characteristics with risk of hospital readmission with a second enteric infection within 12 months of a first hospital admission with an enteric infection. (DOCX 86 kb) [file 12887_2018_1079_MOESM4_ESM.docx]

# Additional file 4. Associations of demographic and illness characteristics with risk of hospital readmission with a second enteric infection within 12 months of a first hospital admission with an enteric infection.

|  | **Enteric infection readmission within 12 months** | | | | |
| --- | --- | --- | --- | --- | --- |
|  | **n (row %)** | | **Multivariable** |  |  |
|  | **Yes** | **No** | **odds ratio** |  |  |
| **Variable** | **n = 449** | **n = 6,460** | **(95% CI)** | **Forest Plot** | ***P*-value** |
| **Demographic characteristics** | | | | | |
| **Age** |  |  |  |  |  |
| Less than 6 months | 157 (9) | 1,598 (91) | **1.53 (1.24-1.87)** |  | **<0.001** |
| 6 to 23 months old | 292 (6) | 4,862 (94) | 1.00 |  |  |
| **Gender** |  |  |  |  |  |
| Male | 263 (7) | 3,408 (93) | **1.25 (1.03-1.52)** |  | **0.03** |
| Female | 186 (6) | 3,052 (94) | 1.00 |  |  |
| **Ethnicity*** |  |  |  |  |  |
| Pacific | 63 (7) | 778 (93) | 1.16 (0.84-1.58) |  | 0.36 |
| Māori | 108 (6) | 1,652 (94) | 0.95 (0.73-1.22) |  | 0.68 |
| Asian | 34 (7) | 461 (93) | 1.07 (0.72-1.54) |  | 0.72 |
| Other | 11 (7) | 157 (93) | 1.11 (0.56-2.00) |  | 0.74 |
| European | 233 (6) | 3,399 (94) | 1.00 |  |  |
| **Household deprivation^†^** |  |  |  |  |  |
| Dep 9 & 10 (most deprived) | 164 (7) | 2,230 (93) | 1.05 (0.75-1.51) |  | 0.77 |
| Dep 7 & 8 | 99 (6) | 1,520 (94) | 0.93 (0.65-1.33) |  | 0.67 |
| Dep 5 & 6 | 68 (6) | 1,142 (94) | 0.87 (0.60-1.28) |  | 0.48 |
| Dep 3 & 4 | 68 (8) | 813 (92) | 1.22 (0.84-1.80) |  | 0.30 |
| Dep 1 & 2 (least deprived) | 50 (6) | 736 (94) | 1.00 |  |  |
| **Season of first admission**^ǂ^ |  |  |  |  |  |
| Autumn | 96 (7) | 1,267 (93) | 1.00 (0.75-1.35) |  | 0.98 |
| Winter | 114 (7) | 1,469 (93) | 1.06 (0.80-1.41) |  | 0.70 |
| Spring | 144 (10) | 1,257 (90) | 0.81 (0.62-1.06) |  | 0.12 |
| Summer | 95 (4) | 2,467 (94) | 1.00 |  |  |
| **Illness characteristics** | | | | | |
| **Presence of complex chronic condition** |  |  |  |  |  |
| Yes | 18 (21) | 66 (79) | **32** |  | **<0.001** |
| No | 431 (6) | 6,394 (94) | 1.00 |  |  |
| **Length of stay** |  |  |  |  |  |
| ≥3 days | 205 (7) | 2,631 (93) | 1.17 (0.96-1.42) |  | 0.11 |
| 2 days or less | 244 (6) | 3,829 (94) | 1.00 |  |  |
|  | | | | | |
| * Ethnicity not stated, n = 13  ^†^ Area-level socio-economic deprivation was measured using the NZ Index of Deprivation (NZDep06), grouped into quintiles [[13](#_ENREF_13)]. Data were missing for 137 (0.2%) children.  ^ǂ^ Autumn = March to May; Winter = June to August; Spring = September to November; Summer = December to February.  CI – confidence interval | | | | | |
