# Supplementary material for: Hospital readmissions with acute infectious diseases in New Zealand children < 2 years of age
Source: BMC Pediatr. 2018 Mar 5;18:98. doi: 10.1186/s12887-018-1079-x (PMC5838880; doi:10.1186/s12887-018-1079-x)
Supplement: Supplementary file 5 — Associations of demographic and illness characteristics with risk of hospital readmission with a second skin and soft tissue infection within 12 months of a first hospital admission with a skin and soft tissue infection. (DOCX 91 kb) [file 12887_2018_1079_MOESM5_ESM.docx]

# Additional file 5. Associations of demographic and illness characteristics with risk of hospital readmission with a second skin and soft tissue infection within 12 months of a first hospital admission with a skin and soft tissue infection.

|  | **Skin and soft tissue infection readmission within 12 months** | | | | |
| --- | --- | --- | --- | --- | --- |
|  | **n (row %)** | | **Multivariable** |  |  |
|  | **Yes** | **No** | **odds ratio** |  |  |
| **Variable** | **n = 205** | **n = 3005** | **(95% CI)** | **Forest Plot** | ***P*-value** |
| **Demographic characteristics** | | | | | |
| **Age** |  |  |  |  |  |
| Less than 6 months | 50 (5) | 898 (95) | 0.80 (0.56-1.10) |  | 0.17 |
| 6 to 23 months old | 155 (7) | 2,107 (93) | 1.00 |  |  |
| **Gender** |  |  |  |  |  |
| Male | 89 (6) | 1,498 (94) | 0.78 (0.59-1.04) |  | 0.09 |
| Female | 116 (7) | 1,507 (93) | 1.00 |  |  |
| **Ethnicity*** |  |  |  |  |  |
| Pacific | 59 (8) | 701 (92) | 1.41 (0.92-2.19) |  | 0.12 |
| Māori | 87 (6) | 1,269 (94) | 1.15 (0.77-1.73) |  | 0.50 |
| Asian | 13 (9) | 132 (91) | 1.81 (0.92-3.37) |  | 0.08 |
| Other | 0 (0) | 43 (100) | - |  | - |
| European | 46 (5) | 849 (95) | 1.00 |  |  |
| **Household deprivation^†^** |  |  |  |  |  |
| Dep 9 & 10 (most deprived) | 102 (7) | 1,375 (93) | 0.95 (0.54-1.77) |  | 0.88 |
| Dep 7 & 8 | 48 (6) | 698 (94) | 0.93 (0.52-1.75) |  | 0.81 |
| Dep 5 & 6 | 27 (6) | 406 (94) | 0.93 (0.49-1.82) |  | 0.83 |
| Dep 3 & 4 | 12 (4) | 280 (96) | 0.62 (0.28-1.34) |  | 0.22 |
| Dep 1 & 2 (least deprived) | 16 (6) | 234 (94) | 1.00 |  |  |
| **Season of first admission**^ǂ^ |  |  |  |  |  |
| Autumn | 52 (6) | 777 (94) | 1.04 (0.70-1.54) |  | 0.84 |
| Winter | 53 (7) | 723 (93) | 1.17 (0.79-1.74) |  | 0.43 |
| Spring | 46 (7) | 657 (93) | 1.13 (0.75-1.70) |  | 0.56 |
| Summer | 54 (2) | 2,987 (98) | 1.00 |  |  |
| **Illness Characteristics** | | | | | |
| **Presence of complex chronic condition** |  |  |  |  |  |
| Yes | 0 (0) | 18 (100) | - |  | - |
| No | 205 (6) | 2,987 (94) | 1.00 |  |  |
| **Length of stay** |  |  |  |  |  |
| ≥3 days | 157 (7) | 2,230 (93) | 1.14 (0.83-1.62) |  | 0.42 |
| 2 days or less | 48 (6) | 775 (94) | 1.00 |  |  |
|  | | | | | |
| * Ethnicity not stated, n = 11  ^†^ Area-level socio-economic deprivation was measured using the NZ Index of Deprivation (NZDep06), grouped into quintiles [[13](#_ENREF_13)]. Data were missing for 137 (0.2%) children.  ^ǂ^ Autumn = March to May; Winter = June to August; Spring = September to November; Summer = December to February.  CI – confidence interval | | | | | |
