# Supplementary material for: Hospital readmissions with acute infectious diseases in New Zealand children < 2 years of age
Source: BMC Pediatr. 2018 Mar 5;18:98. doi: 10.1186/s12887-018-1079-x (PMC5838880; doi:10.1186/s12887-018-1079-x)
Supplement: Supplementary file 6 — Associations of demographic and illness characteristics with risk of hospital readmission with a second UTI within 12 months of a first hospital admission with a UTI. (DOCX 75 kb) [file 12887_2018_1079_MOESM6_ESM.docx]

# Additional file 6. Associations of demographic and illness characteristics with risk of hospital readmission with a second UTI within 12 months of a first hospital admission with a UTI.

|  | **Urinary tract infection readmission within 12 months** | | | | |
| --- | --- | --- | --- | --- | --- |
|  | **n (row %)** | | **Multivariable** |  |  |
|  | **Yes** | **No** | **odds ratio** |  |  |
| **Variable, n(%)** | **n = 101** | **n = 1725** | **(95% CI)** | **Forest Plot** | ***P*-value** |
| **Demographic characteristics** | | | | | |
| **Age** |  |  |  |  |  |
| Less than 6 months | 61 (6) | 968 (94) | 1.03 (0.66-1.61) |  | 0.91 |
| 6 to 23 months old | 40 (5) | 757 (95) | 1.00 |  |  |
| **Gender** |  |  |  |  |  |
| Male | 54 (6) | 850 (94) | 1.08 (0.70-1.65) |  | 0.73 |
| Female | 47 (5) | 875 (95) | 1.00 |  |  |
| **Ethnicity*** |  |  |  |  |  |
| Pacific | 20 (6) | 322 (94) | 0.98 (0.53-1.77) |  | 0.95 |
| Māori | 22 (6) | 335 (94) | 1.00 (0.56-1.73) |  | 1.00 |
| Asian | 9 (5) | 183 (95) | 0.80 (0.36-1.61) |  | 0.56 |
| Other | 1 (3) | 32 (97) | 0.60 (0.03-2.96) |  | 0.59 |
| European | 49 (5) | 851 (95) | 1.00 |  |  |
| **Household deprivation^†^** |  |  |  |  |  |
| Dep 9 & 10 (most deprived) | 36 (6) | 579 (94) | 1.19 (0.57-2.62) |  | 0.65 |
| Dep 7 & 8 | 32 (7) | 398 (93) | 1.51 (0.76-3.23) |  | 0.25 |
| Dep 5 & 6 | 13 (4) | 280 (96) | 0.87 (0.38-2.03) |  | 0.74 |
| Dep 3 & 4 | 8 (3) | 247 (97) | 0.62 (0.23-1.56) |  | 0.31 |
| Dep 1 & 2 (least deprived) | 11 (5) | 216 (95) | 1.00 |  |  |
| **Season of first admission**^ǂ^ |  |  |  |  |  |
| Autumn | 23 (5) | 458 (95) | 0.84 (0.47-1.48) |  | 0.54 |
| Winter | 24 (6) | 395 (94) | 1.02 (0.57-1.79) |  | 0.96 |
| Spring | 26 (6) | 418 (94) | 0.93 (0.53-1.63) |  | 0.79 |
| Summer | 28 (6) | 454 (94) | 1.00 |  |  |
| **Illness Characteristics** | | | | | |
| **Presence of complex chronic condition** |  |  |  |  |  |
| Yes | 8 (18) | 36 (82) | **3.49 (1.45-7.51)** |  | **0.007** |
| No | 93 (5) | 1,689 (95) | 1.00 |  |  |
| **Length of stay** |  |  |  |  |  |
| ≥3 days | 85 (6) | 1,290 (94) | 1.70 (0.99-3.09) |  | 0.05 |
| 2 days or less | 16 (4) | 435 (96) | 1.00 |  |  |
|  | | | | | |
| * Ethnicity not stated, n = 2  ^†^ Area-level socio-economic deprivation was measured using the NZ Index of Deprivation (NZDep06), grouped into quintiles [[13](#_ENREF_13)]. Data were missing for 6 (0.3%) children.  ^ǂ^ Autumn = March to May; Winter = June to August; Spring = September to November; Summer = December to February.  CI – confidence interval | | | | | |
